# Cellular identity and Ca^2+^ signaling activity of the non-reproductive GnRH system in the *Ciona intestinalis* type A (*Ciona robusta*) larva

**DOI:** 10.1038/s41598-020-75344-7

**Published:** 2020-10-29

**Authors:** Nanako Okawa, Kotaro Shimai, Kohei Ohnishi, Masamichi Ohkura, Junichi Nakai, Takeo Horie, Atsushi Kuhara, Takehiro G. Kusakabe

**Affiliations:** 1 Department of Biology, Faculty of Science and Engineering, Konan University, 8-9-1 Okamoto, Higashinada-ku, Kobe, 658-8501 Japan; 2 Institute for Integrative Neurobiology, Graduate School of Natural Science, Konan University, 8-9-1 Okamoto, Higashinada-ku, Kobe, 658-8501 Japan; 3 Brain and Body System Science Institute, Saitama University, Saitama, Japan; 4 Division of Oral Physiology, Department of Oral Function and Morphology, Tohoku University Graduate School of Dentistry, 4-1 Seirhyo-machi, Aoba-ku, Sendai 980-8575 Japan; 5 Shimoda Marine Research Center, University of Tsukuba, Shimoda, 415-0025 Japan

**Keywords:** Evolutionary developmental biology, Peptide hormones, Glial biology, Motor control, Neural circuits

## Abstract

Tunicate larvae have a non-reproductive gonadotropin-releasing hormone (GnRH) system with multiple ligands and receptor heterodimerization enabling complex regulation. In *Ciona intestinalis* type A larvae, one of the *gnrh* genes, *gnrh2*, is conspicuously expressed in the motor ganglion and nerve cord, which are homologous structures to the hindbrain and spinal cord, respectively, of vertebrates. The *gnrh2* gene is also expressed in the proto-placodal sensory neurons, which are the proposed homologue of vertebrate olfactory neurons. Tunicate larvae occupy a non-reproductive dispersal stage, yet the role of their GnRH system remains elusive. In this study, we investigated neuronal types of *gnrh2*-expressing cells in *Ciona* larvae and visualized the activity of these cells by fluorescence imaging using a calcium sensor protein. Some cholinergic neurons and dopaminergic cells express *gnrh2*, suggesting that GnRH plays a role in controlling swimming behavior. However, none of the *gnrh2*-expressing cells overlap with glycinergic or GABAergic neurons. A role in motor control is also suggested by a relationship between the activity of *gnrh2*-expressing cells and tail movements. Interestingly, *gnrh2*-positive ependymal cells in the nerve cord, known as a kind of glia cells, actively produced Ca^2+^ transients, suggesting that active intercellular signaling occurs in the glia cells of the nerve cord.

## Introduction

Gonadotropin-releasing hormone (GnRH) is a key regulator of reproductive functions in vertebrates^1,2^. GnRH has also been suggested to play non-reproductive roles in the nervous system and during development^3–9^. Compared to its reproductive roles, however, the non-reproductive roles of GnRH are less well understood.

Tunicates are the sister group of vertebrates^10,11^. A conspicuous non-reproductive GnRH system has been reported in the larva of the sessile tunicate *Ciona intestinalis* type A (also called *Ciona robusta*)^12,13^. Six GnRH peptides and four receptors are encoded by the *Ciona* genome^14–18^. In the *Ciona* larva, the GnRH genes are strikingly expressed in the central nervous system (CNS) through the entire antero-posterior body axis^12^. Correspondingly, the GnRH receptor genes are specifically expressed in the tissues and organs located along the CNS, namely the notochord, the tail muscle, and the epidermal sensory neurons^12^. One of the *Ciona gnrh* genes, *gnrh2*, is conspicuously expressed in the motor ganglion and nerve cord of the larva, which are homologous structures to the hindbrain and spinal cord, respectively, of vertebrates. The *gnrh2* gene is also expressed in the proto-placodal sensory neurons, which are the proposed homologue of vertebrate olfactory neurons^19^. *Ciona* GnRH has been suggested to play a pivotal role in the control of metamorphosis^13^. Considering the complex and well-developed nature of the larval GnRH system in *Ciona*, GnRH may play diverse and important roles in developmental and physiological processes in *Ciona* larvae. To date, however, the roles of the *Ciona* GnRH system remain elusive.

In this study, we investigated neuronal types of *gnrh2*-expressing cells in the *Ciona* larva and visualized the activity of these cells by fluorescence imaging using a calcium sensor protein. Some cholinergic motor neurons as well as unique cholinergic cells along the nerve cord were found to express *gnrh2*, suggesting that GnRH plays a role in the control of swimming behavior. By contrast, none of the *gnrh2*-expressing cells overlapped with glycinergic or GABAergic neurons. A role in motor control was also suggested by a relationship between the activity of some *gnrh2*-expressing cells and tail movements. Interestingly, *gnrh2*-positive ependymal cells in the nerve cord, known as a kind of glia cells, produced Ca^2+^ transients, suggesting that active intercellular signaling occurs in in the glia cells of the nerve cord.

## Results

### *Gnrh2* is expressed in proto-placode-derived sensory neurons and caudal glial ependymal cells

**Figure 1:**
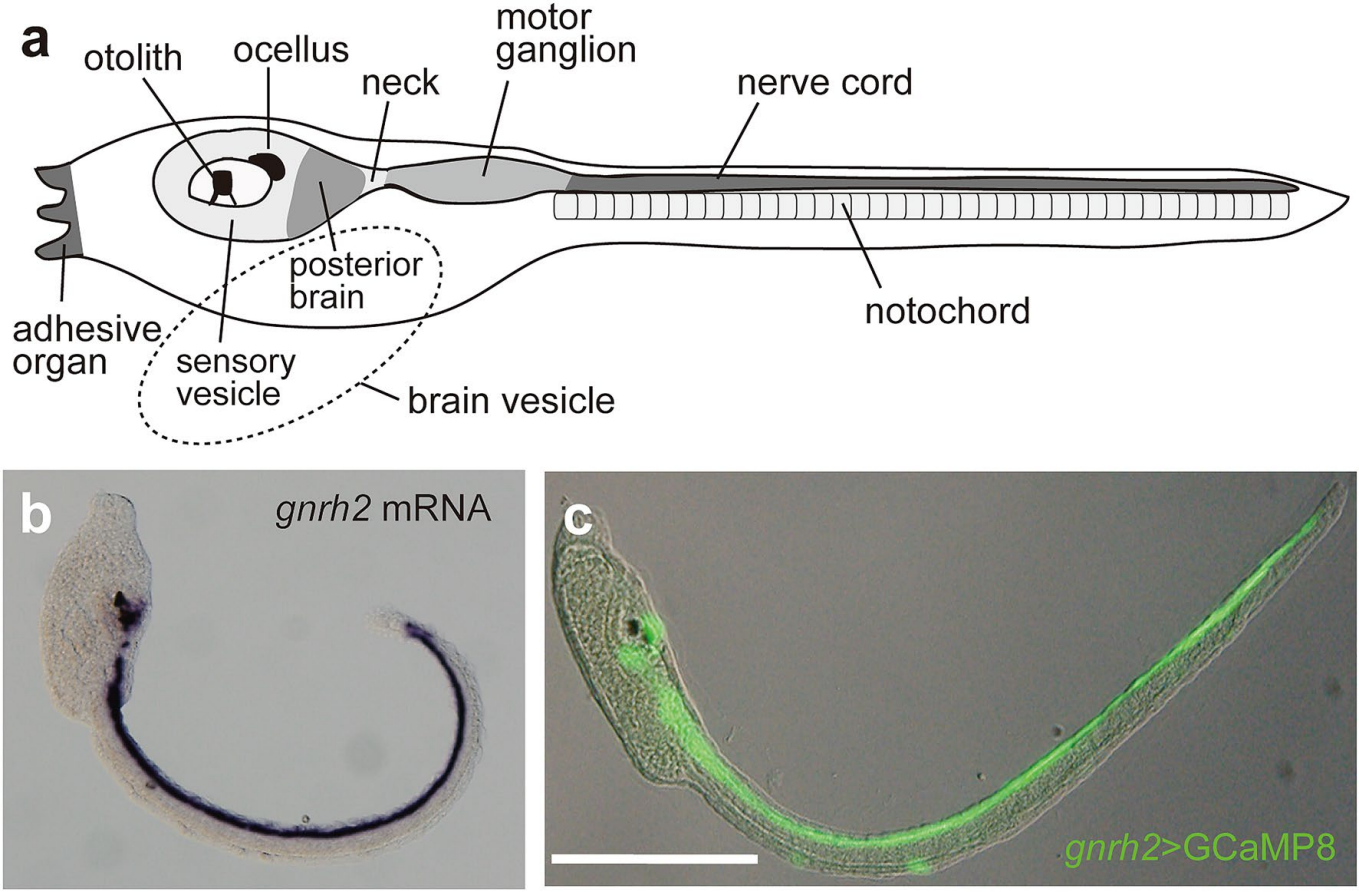
Expression patterns of *gnrh2* in the *Ciona* larva. (**a**) Schematic diagram showing the CNS of the larva. (**b**) Localization of *gnrh2* mRNA visualized by in situ hybridization. (**c**) Immunofluorescent localization of G-CaMP8 expressed under the control of the *cis*-regulatory region of *gnrh2* (*gnrh2* > *g-camp8*) in a larva at 21 h post-fertilization (hpf). The expression patterns of *g-camp8* were consistent with the endogenous *gnrh2* expression in all larvae examined (n = 45). The results for eight other larvae are shown in Supplementary Figure S1. Scale bar, 200 µm.

**Figure 2:**
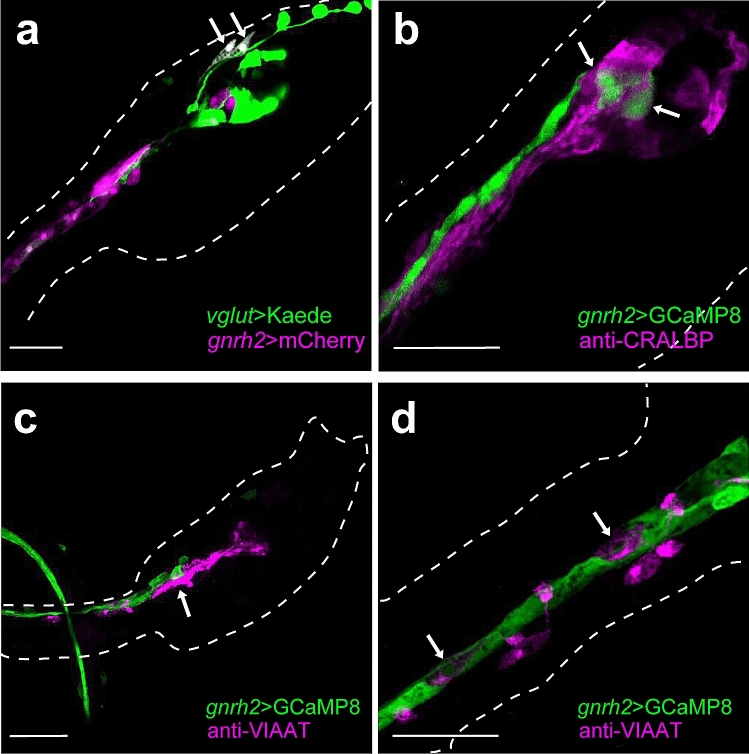
Immunohistochemical identification of types of cells expressing *gnrh2* in the *Ciona* larva at 21 hpf. (**a**) Glutamatergic neurons and *gnrh2*-expressing cells were labeled with Kaede (*green*) and mCherry (*magenta*), respectively. The proto-placode-derived sensory neurons (aATENs; *arrows*) were shown to express *gnrh2* (6 of 6 larvae showed the expression; four additional examples are shown in Supplementary Figure S2). (**b**) CRALBP-positive cells (*magenta*) were not overlapped with *gnrh2*-expressing cells (*green*) (none of 16 larvae showed overlapped expression; four additional examples are shown in Supplementary Figure S3). *Arrows* indicate *gnrh2*-expressing cells in the brain vesicle. (**c**, **d**) GABAergic/glycinergic neurons were visualized by immunostaining with anti-VIAAT antibody (*magenta*). VIAAT-positive cells (*magenta*) were not overlapped with *gnrh2*-expressing cells (*green*) (none of 17 larvae showed overlapped expression; four additional examples are shown in Supplementary Figure S3). *Arrows* in (**c**) indicate GABAergic/glycinergic neurons in the motor ganglion. *Arrows* in (**d**) indicate VIAAT-positive ACINs. (**a**) Projection of 7 serial optical sections taken at 0.60 µm intervals. (**b**) Projection of 8 serial optical sections taken at 0.60 µm intervals. (**c**) Projection of 7 serial optical sections taken at 0.60 µm intervals. (**d**) Projection of 3 serial optical sections taken at 0.60 µm intervals. Scale bars, 30 µm.

**Figure 3:**
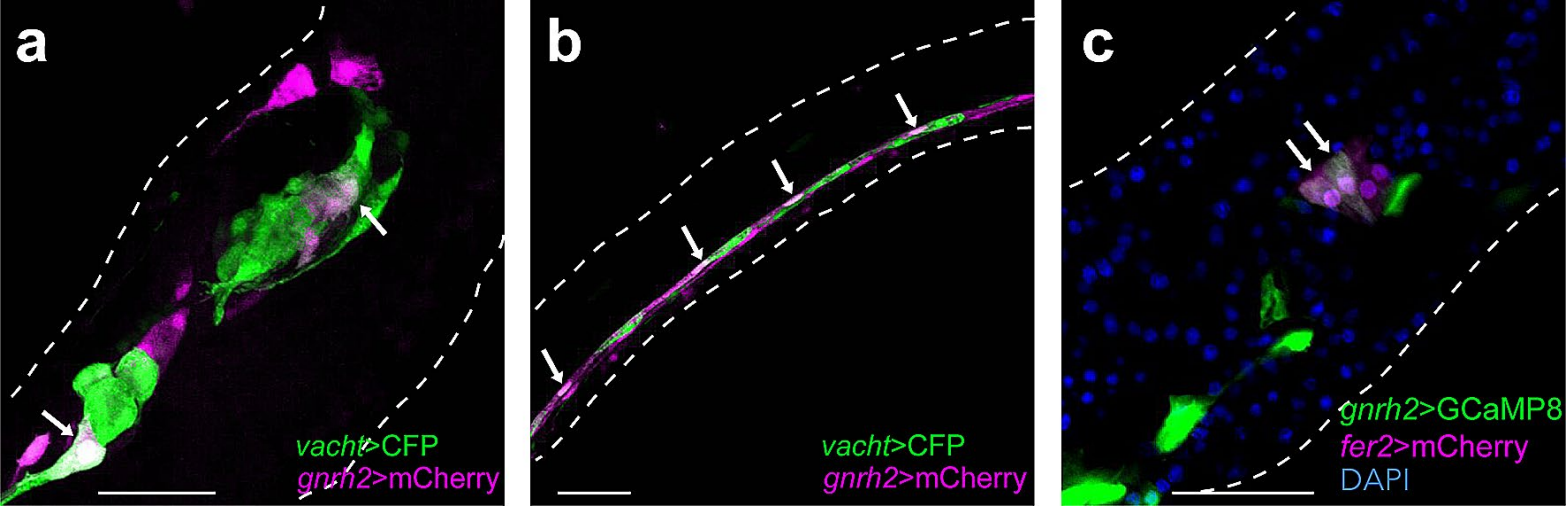
Some cholinergic and dopaminergic neurons express *gnrh2*. (**a**, **b**) Cholinergic neurons and *gnrh2*-exressing cells were labeled with CFP (*green*) and mCherry (*magenta*), respectively. *Arrows* indicate cells that co-expressed both markers. In the motor ganglion, overlapped expression of two reporters was observed in 10 of 13 larvae (two additional examples are shown in Supplementary Figure S4). In the nerve cord, overlapped expression of two reporters was observed in 4 of 5 larvae (two additional examples are shown in Supplementary Figure S4). (**c**) Dopaminergic cells and *gnrh2*-expressing cells were labeled with mCherry (*magenta*) and G-CaMP8 (*green*), respectively. Some dopaminergic cells were also labeled with G-CaMP8 (*arrows*). Overlapped expression of mCherry and G-CaMP8 was observed in 10 of 16 larvae. Two additional examples are shown in Supplementary Figure S5. (**a**) Projection of 10 serial optical sections taken at 0.60 µm intervals. (**b**) Projection of 7 serial optical sections taken at 0.60 µm intervals. (**c**) Confocal image of a single optical section. Scale bars, 30 µm.

The 4.3-kb upstream region of *gnrh2* connected with a fluorescence reporter can recapitulate the expression patterns of *gnrh2 *^12^ (Fig. 1; Supplementary Fig. S1). This upstream region was used to transiently express mCherry and G-CaMP8 in cells expressing *gnrh2* (Figs. 1, 2 and 3). Cell types were identified by double fluorescent staining of larvae with cell type-specific markers. The expression of electroporated transgenes displayed some mosaicism in *Ciona* larvae. Expression patterns of the reporter gene in each animal usually represent parts of the regions where the promoter can be activated, but the pattern obtained in dozens of larvae in at least three independent experiments per transgene was consistent. The mosaicism allowed us to visualize different populations of *gnrh2*-expressing cells. The number of larvae examined for each analysis are described in the figure legends, and additional data are presented in the Supplementary Information.

First, we examined whether *gnrh2*-expressing cells include glutamatergic neurons. In the *Ciona* larva, glutamate is a major neurotransmitter in the peripheral sensory neurons and photoreceptor cells^20^. Some interneurons in the posterior brain are also glutamatergic^20^. As previously reported^19^, proto-placode-derived *gnrh2*-expressing epidermal neurons (aATENs) are glutamatergic (Fig. 2a; Supplementary Fig. S2). In the CNS, glutamatergic neurons located at the ventral region of the posterior brain vesicle seem to express *gnrh2* (Supplementary Fig. S2).

Next, we examined whether any of the GABAergic/glycinergic neurons express *gnrh2* using vesicular inhibitory amino acid transporter (VIAAT) as a marker. None of the VIAAT-positive cells overlapped with the reporter expression under the control of the *gnrh2 cis*-regulatory region (Fig. 2c and d; Supplementary Fig. S3). In the anterior tail region, there are two pairs of VIAAT-positive neurons called anterior caudal inhibitory neurons (ACINs), which align with glial ependymal cells in the lateral wall of the anterior nerve cord^21,22^. Our result suggests that the ACINs do not express *gnrh2*, whereas the lateral ependymal cells express *gnrh2* (Fig. 2d). The *gnrh2* expression in the lateral wall ependymal cells of the nerve cord is consistent with the in situ hybridization data previously reported^12^.

Cellular retinaldehyde-binding protein (CRALBP) is specifically localized in the glial ependymal cells in the brain vesicle and the motor ganglion^23,24^. In our immunohistochemical analysis, CRALBP-positive cells were never overlapped with *gnrh2*-epxressing cells (Fig. 2b; Supplementary Fig. S3). Thus, in contrast to the conspicuous *gnrh2* expression in the ependymal cells of the nerve cord, *gnrh2* does not seem to be expressed in the ependymal cells in the brain vesicle and the motor ganglion.

### Some cholinergic and dopaminergic neurons express *gnrh2*

Acetylcholine is a major neurotransmitter at the neuromuscular junctions of the *Ciona* larva^21,25^. Cholinergic neurons were visualized by a fluorescence protein expressed under the control of the *cis*-regulatory region of the *vacht* gene^25^. *Gnrh2*-expressing neurons were shown to be cholinergic both in the brain vesicle and the motor ganglion (Fig. 3a; Supplementary Fig. S4).

The caudal part of the CNS (nerve cord) mainly consists of non-neuronal ependymal cells^26^. The nerve cord also contains two types of neurons: ACINs and bilateral pairs of cholinergic caudal neurons^21^. Some of these cholinergic caudal neurons seem to express *gnrh2* (Fig. 3b; Supplementary Fig. S4).

Another neurotransmitter that controls the swimming of *Ciona* tadpoles is dopamine^27^. Dopaminergic neurons are present in the brain vesicle^27–29^. Dopaminergic neurons were labeled with mCherry expressed under the control of the *cis*-regulatory region of the dopaminergic cell-specific gene *fer2*^27,29^ (previously described as *Ptf1a*; see Gyoja & Satoh^30^ for the orthologous families of the bHLH transcription factors). Double fluorescence imaging of dopaminergic neurons and *gnrh2*-expressing cells suggested that some dopaminergic neurons express *gnrh2* (Fig. 3c; Supplementary Fig. S5).

### Active Ca^2+^ transients in aATENs and *gnrh2*-expressing cells of the posterior CNS

**Figure 4:**
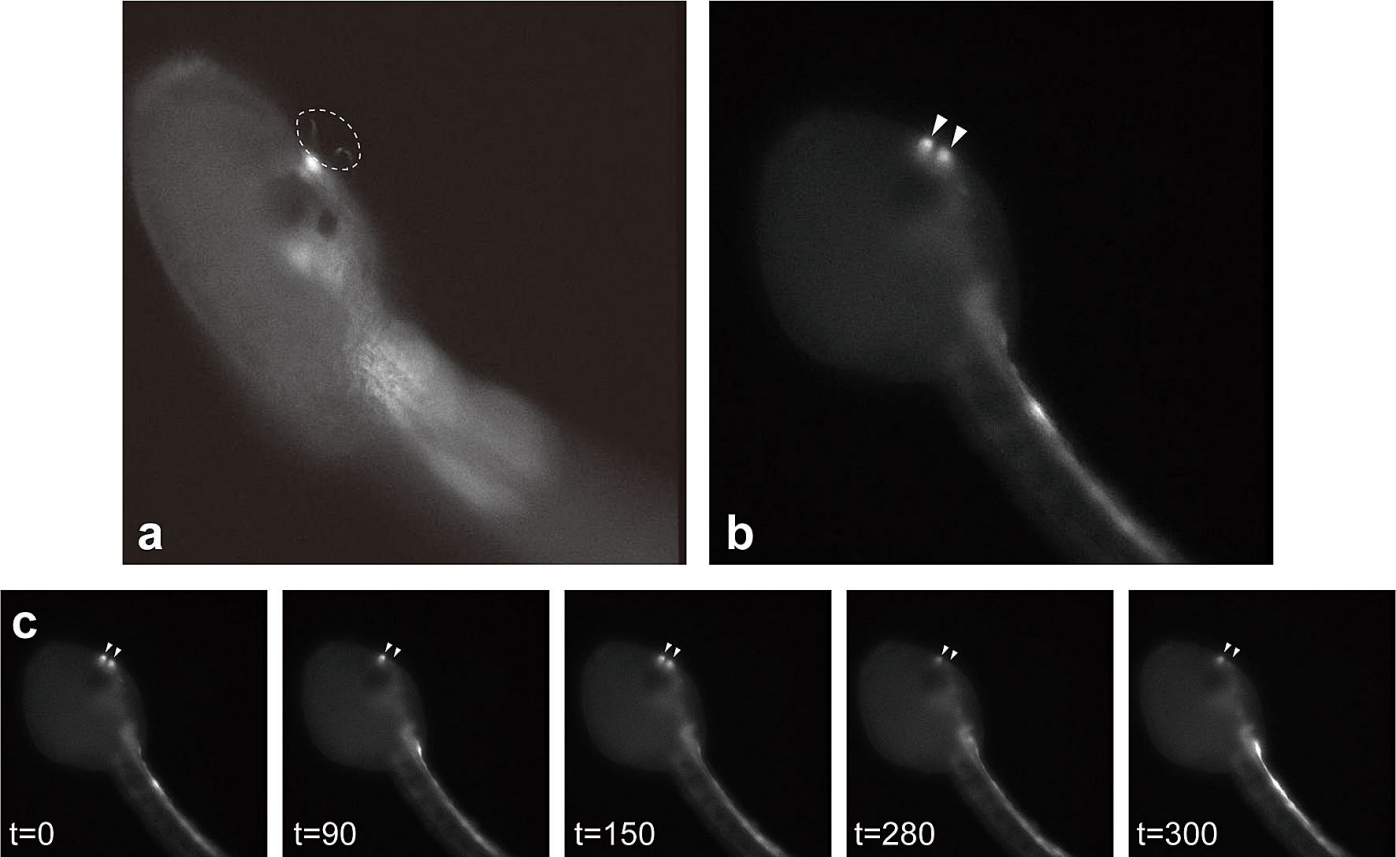
Calcium imaging of *gnrh2*-expressing chemosensory neurons. Fluorescence images of *Ciona* larvae expressing G-CaMP8 in the chemosensory aATENs. (a) The putative sensory cilum of each aATEN was labeled with G-CaMP8 fluorescence (dotted circle). (**b**, **c**) An example of a larva showing dynamic Ca^2+^ transients in a pair of aATENs (*arrowheads*) at 19 hpf. (**c**) Representative images of the larva recorded at the times indicated (in seconds). Serial images of the larva shown in (**b**, **c**) are shown in Movie S1. Quantitative analysis of the imaging data is shown in Supplementary Figure S6.

**Figure 5:**
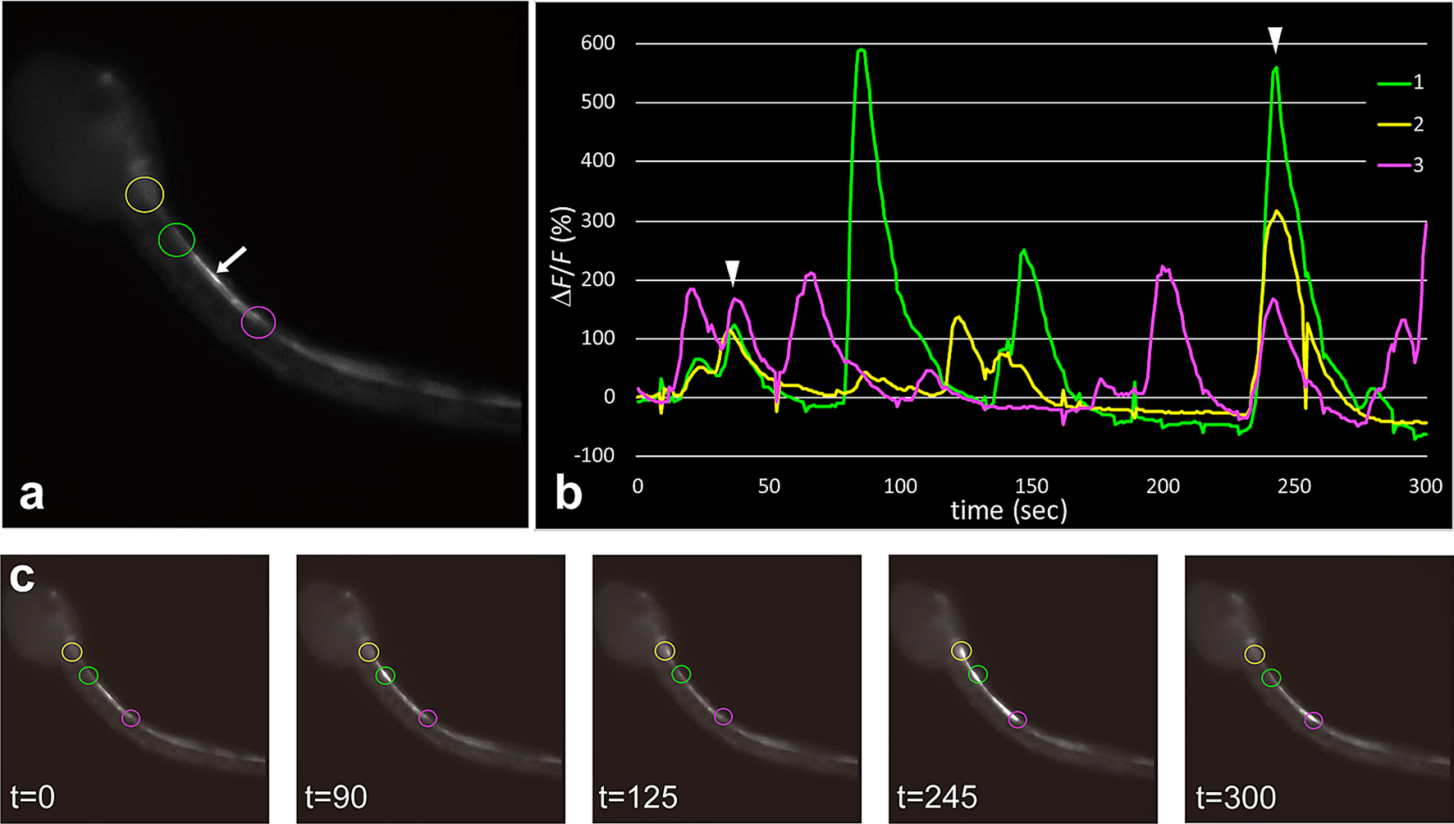
Interconnection between *gnrh2*-expressing cells in the larval CNS. (**a**) Fluorescence image of a larva at 19 hpf, showing G-CaMP8 fluorescence in the motor ganglion and the anterior nerve cord. (**b**) The graph shows the temporal patterns of fluorescence intensity at the three sites indicated by circles in (**a**). The colors of the lines correspond to the sites indicated by circles in the respective colors. The Ca^2+^ transients occurred independently of each other, but sometimes occurred at the same time (*arrowheads*). (**c**) Representative images of the larva recorded at the times indicated (in seconds). Serial images of the larva shown in (A) are shown in Movie S2.

**Figure 6:**
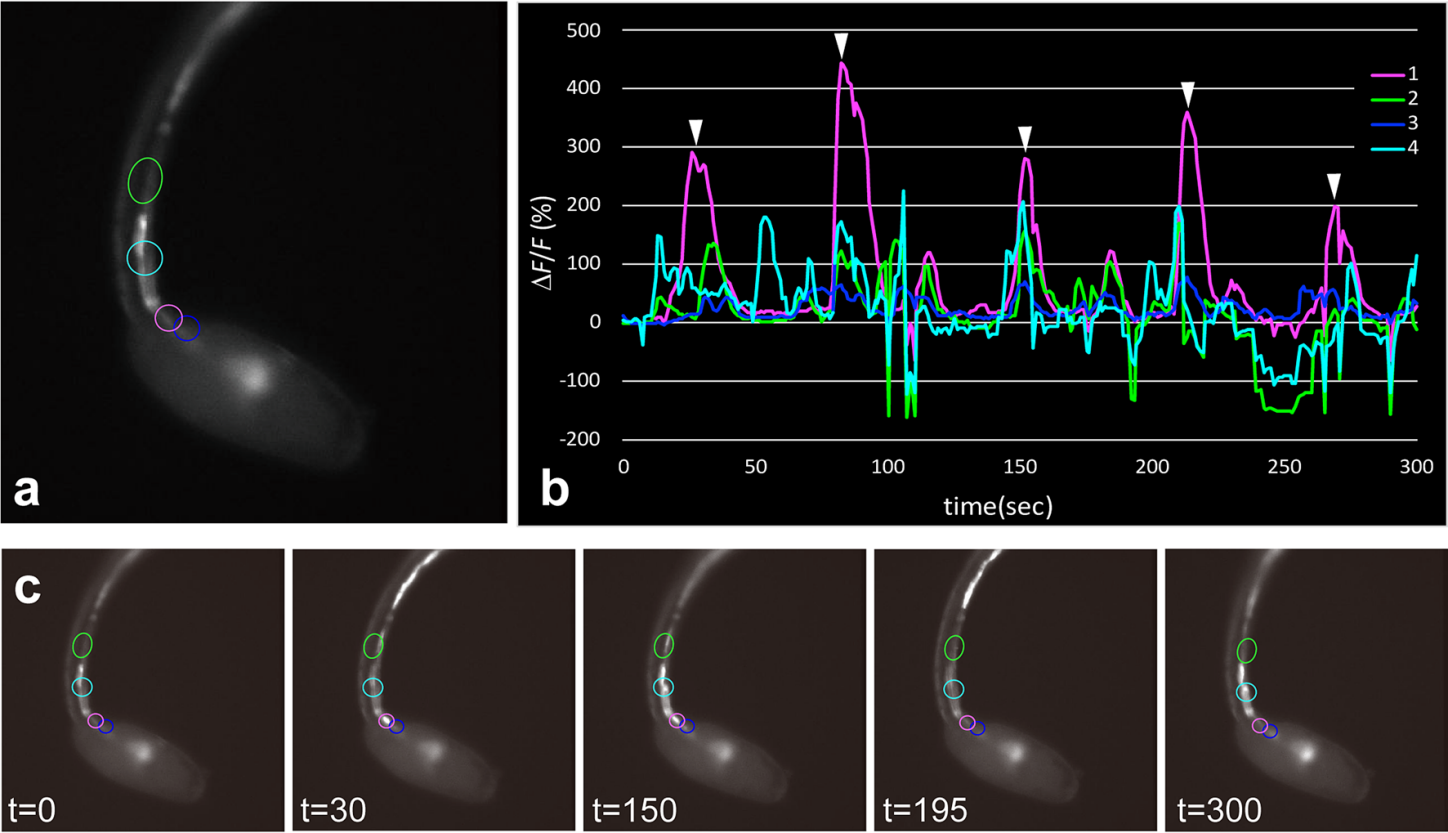
Periodic oscillation of Ca^2+^ transients in a *gnrh2*-expressing cell in the motor ganglion. (**a**) Fluorescence image of a larva at 20 hpf, showing G-CaMP8 fluorescence in the motor ganglion and the anterior nerve cord. (**b**) The graph shows the temporal patterns of fluorescence intensity at the four sites indicated by circles in (**a**). The colors of the lines correspond to the sites indicated by circles in the respective colors. Ca^2+^ spikes were periodically observed at regular intervals in the cell indicated by the magenta circle in (**a**) (*arrowheads*). (**c**) Representative images of the larva recorded at the times indicated (in seconds). Serial images of the larva shown in (**a**) are shown in Movie S3.

G-CaMP8^31^ was used to monitor temporal changes in intracellular Ca^2+^ in *gnrh2*-expressing cells. We analyzed 51 larvae derived from 16 independent transfections (each transfection gave 1–5 larvae to the Ca^2+^ imaging analysis). Among the 51 larvae examined, 24, 40, and 41 larvae showed Ca^2+^ transients in aATENs, the motor ganglion, and the nerve cord, respectively. Active Ca^2+^ transients were observed in aATENs, the motor ganglion, and the caudal nerve cord (Figs. 4, 5, 6; Movies S1–S3). The larva contains two aATENs, and each has a sensory cilium^19^ (Fig. 4a,b). Both aATENs showed Ca^2+^ transients (Fig. 4c; Movie S1; Supplementary Fig. S6). In the larva shown in Fig. 4c, the activities of the two aATENs showed a moderate positive correlation (Supplementary Fig. S6).

Some *gnrh2*-expressing cells at the posterior part of the motor ganglion exhibited active Ca^2+^ transients (Figs. 5 and 6). Periodic Ca^2+^ transients of a *gnrh2*-expressing neuron in the motor ganglion were observed at 20 h post-fertilization (hpf) (*arrowheads* in Fig. 6b). Similar periodic Ca^2+^ transients in the motor ganglion were observed in at least three additional larvae at 19–20 hpf. In the tail region, Ca^2+^ transients were observed through the entire length of the nerve cord (Figs. 5 and 6). Both cholinergic neurons and ependymal cells showed Ca^2+^ transients in the tail nerve cord. For example, the narrow cell indicated by an *arrow* in Fig. 5a is presumably a cholinergic neuron. Many cells showing Ca^2+^ transients were block-shaped, which is characteristic of caudal ependymal cells (Fig. 6c).

Ca^2+^ transients were observed at various times in cells located at different sites of the larva (Figs. 5 and 6). However, simultaneous activation of cells at different sites was occasionally observed (similar patterns were observed in at least 7 larvae), as indicated by arrowheads in Figs. 5a and 6b, suggesting the presence of a neural circuit connecting *gnrh2*-expressing cells at different sites.

### Relationship between tail movements and Ca^2+^ transients in the motor ganglion and the anterior nerve cord

**Figure 7:**
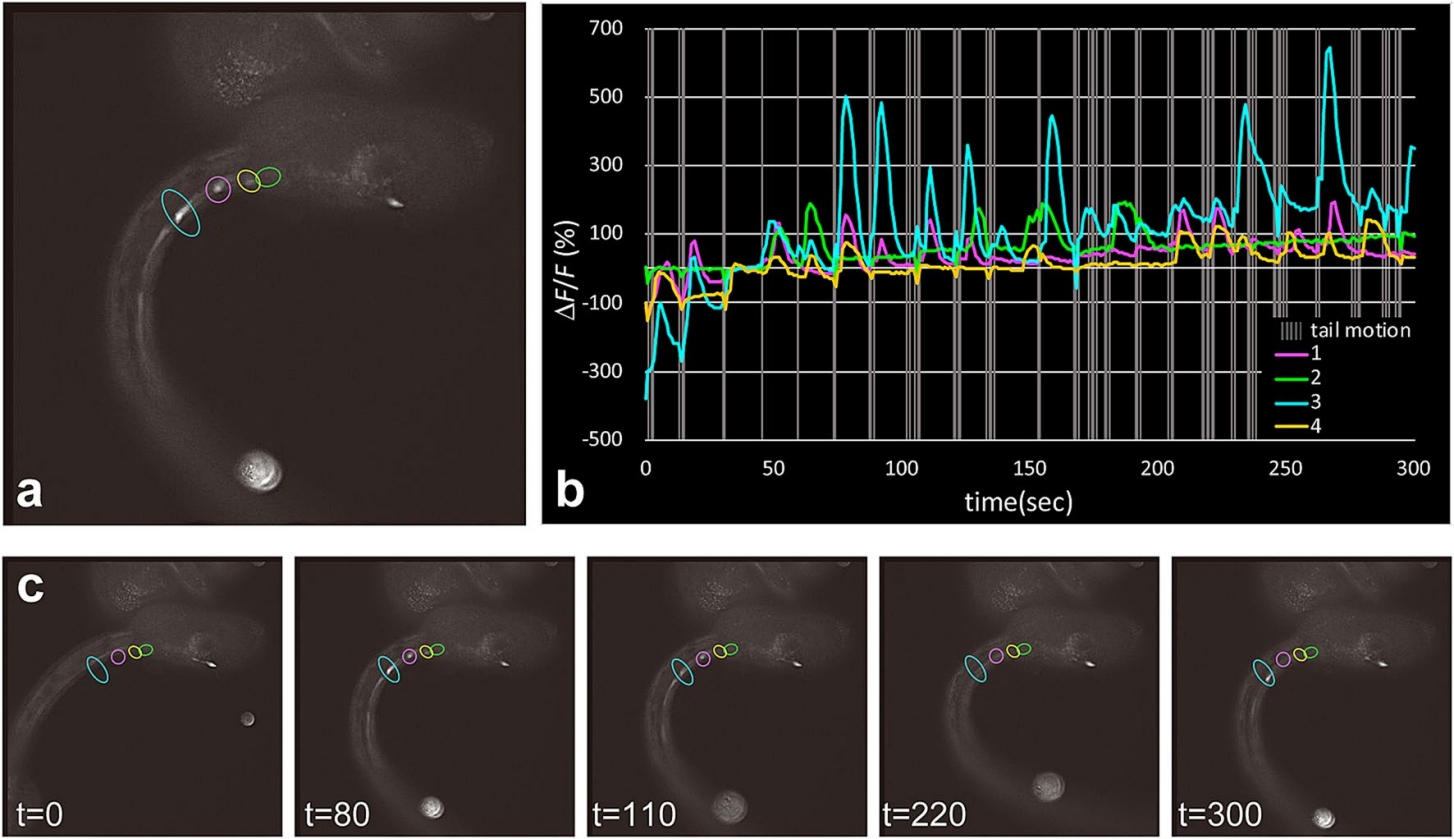
Relationship between Ca^2+^ transients in *gnrh2*-expressing cells and tail movement. (**a**) Fluorescence image of a larva at 19 hpf, showing G-CaMP8 fluorescence in the anterior nerve cord. (**b**) The graph shows the temporal patterns of fluorescence intensity at the four sites encircled by colored lines. The colors of the lines in the graph correspond to the sites encircled by lines of the respective colors. Gray vertical lines indicate the period when the tail was moving. Ca^2+^ transients generally occurred when the tail movement stopped. (**c**) Representative images of the larva recorded at the times indicated (in seconds). Serial images of the larva shown in (**a**) are shown in Movie S4. A statistical evaluation of the relationship between Ca^2+^ transients and tail movement is shown in Supplementary Figure S7.

A neural circuit in the motor ganglion and the anterior nerve cord is thought to control muscle contraction in the tail^21,32,33^. Because active Ca^2+^ transients in *gnrh2*-expressing cells were observed in these regions, we examined the temporal relationship between tail movement and the activity of *gnrh2*-expressing cells. Due to extreme difficulties in obtaining Ca^2+^ imaging data from larvae with a quickly moving tail, we were only able to take serial fluorescence images from the single individual shown in Fig. 7. Ca^2+^ transients were frequently observed when the tail ceased its movement. Thus the tail movement often precedes the Ca^2+^ spike. This pattern can be interpreted as demonstrating that the cessation of tail movement leads to Ca^2+^ transients. This view is consistent with our observation that intracellular Ca^2+^ returned to a basal level before the tail began to move again (Supplementary Fig. S7). These findings suggest the possible involvement of *gnrh2*-expressing cells in the control of swimming behavior.

## Discussion

In this study, we identified cell types of *gnrh2*-expressing cells and visualized their activity in the *Ciona* larva. Previously, the cells expressing GnRH-encoding genes had been only partially identified in *Ciona*. The caudal ependymal cells and the aATENs were reported to express *gnrh2*^12,19^. We confirmed these findings and further identified CNS neurons expressing *gnrh2*.

In the brain vesicle, dopaminergic neurons, glutamatergic neurons located at the posterior ventral region, and a limited number of cholinergic neurons seem to express *gnrh2*. Pharmacological and behavioral analyses have suggested that dopaminergic cells modulate the light-off-induced swimming behavior of *Ciona* larvae^27^. The role of cholinergic neurons in the *Ciona* brain vesicle has not been elucidated. Our present observations are the first to show the heterogeneity of cholinergic neurons in the brain vesicle, and should provide clues for future investigations into the roles of these neurons.

Cholinergic neurons in the motor ganglion have been implicated in the regulation of tail muscle contraction^21,32–34^. Here we show that one subtype of cholinergic neurons in the motor ganglion expresses *gnrh2* (Fig. 3a). These neurons extend axons posteriorly, but it is unclear whether they are motor neurons that directly innervate muscle cells or interneurons that connect to other CNS neurons in the caudal nerve cord. In the nerve cord, another class of cholinergic cells also expresses *gnrh2* (Fig. 3b). One possible role of cholinergic/GnRH neurons in the motor ganglion and the nerve cord may be the control of swimming behavior. These neurons may also play a role in metamorphosis, because GnRH has been suggested to be involved in the regulation of metamorphosis^13^.

Calcium imaging has been applied to studies of *Ciona* development^35–37^. These previous studies focused on Ca^2+^ transients in embryos but not in larvae. The present study is thus the first to report the spatio-temporal patterns of Ca^2+^ transients in larvae of *Ciona*. Our observations included four novel findings: (i) active Ca^2+^ transients in the proto-placode-derived aATENs, (ii) periodic spikes in the motor ganglion, (iii) a relationship between Ca^2+^ transients and tail movements, and (iv) active Ca^2+^ transients in ependymal cells of the nerve cord.

The proto-placode-derived aATENs share morphological and molecular properties with vertebrate olfactory neurons and are thought to be chemosensory cells^19^. However, olfactory receptors have not been identified in *Ciona*, and the chemical cues that stimulate aATENs are not known. Calcium imaging with *gnrh2* > G-CaMP8 could help us search for chemical cues that trigger the activation of aATENs in future studies.

Periodic Ca^2+^ transients observed in the motor ganglion are reminiscent of the spontaneous rhythmic activities observed in the developing nervous systems of vertebrates^38–43^. These periodic neuronal activities are thought to be important for the development of neural circuits in the CNS and the retina^41,44,45^. Similar rhythmic oscillation of Ca^2+^ transients was reported in the developing motor ganglion of the *Ciona* embryo^37^. By contrast, we observed rhythmic Ca^2+^ transients in larvae at 19–20 hpf. The swimming behavior of *Ciona* larvae reveals ontogenic changes; the larvae hatch at 18 hpf (18 °C) and their photo-responsiveness appears within 4 h after hatching^46–48^. Thus, the spontaneous rhythmic Ca^2+^ transients may play an important role in the neural circuit development of *Ciona* larvae.

We observed an association between the tail movements and Ca^2+^ transients in the motor ganglion and the nerve cord. This suggests that *gnrh2*-expressing cells are involved in the control of swimming locomotion. Ca^2+^ transients appeared when the tail stopped moving, and the Ca^2+^ signal was low when the tail was moving (Fig. 7b; Supplementary Fig. S7). In other words, the tail movement precedes the Ca^2+^ spike. This pattern suggests that these *gnrh2*-expressing cells are not motor neurons. In fact, the majority of the cells expressing *gnrh2* in the nerve cord are ependymal cells, and we observed Ca^2+^ transients in ependymal cells. An intriguing possibility is that Ca^2+^ spikes are induced in ependymal cells by muscle contraction or motor axon excitation. If so, the ependymal cells may monitor the activity of muscle or motor neurons. It has been reported that various types of glia cells exhibit Ca^2+^ transients in response to neuronal activities and regulate neuronal functions in vertebrates^49–52^. The ependymal cells of *Ciona* larva may have similar regulatory roles, suggesting a deep evolutionary conservation of glia function between tunicates and vertebrates. Given the simplicity of its nervous system, the *Ciona* larva could serve as a unique model for the study of glia-neuron interaction.

In the present study, however, the observed relationship between the tail motion and the Ca^2+^ transients was largely associative, and the causal relationship and underlying molecular and cellular mechanisms remain unclear. These topics should be addressed by future optogenetic approaches, such as by controlling the activity of specific neurons, glia, and muscle cells by using light-activated ion channels^53^.

In conclusion, the present study revealed the presence of dynamic Ca^2+^ transients of *gnrh2*-expressing cells at various sites in the *Ciona* larva. Our findings suggest a connection between the activity of *gnrh2*-expressing cells and the tail movements of the larva. An important yet unsolved question is whether GnRH2 is involved in these processes. Future studies should address the developmental and physiological roles of *gnrh2*-expressing cells and GnRH peptides based on the findings of this study.

## Methods

### Ethical issues and approval

All animal treatments in this research were carried out in accordance with the Japanese Act on Welfare and Management of Animals (Act No. 105 of October 1, 1973; the latest revision is Act No. 51 of June 2, 2017, effective June 1, 2018). All experimental protocols were approved by the Institutional Animal Care and Use Committees of Konan University, Saitama University, Tohoku University, and the University of Tsukuba.

### Biological materials

Mature adults of *Ciona intestinalis* type A were provided by the Maizuru Fisheries Research Station of Kyoto University and by the Misaki Marine Biological Station of the University of Tokyo through the National Bio-Resource Project of the Ministry of Education, Culture, Sports, Science and Technology of Japan (MEXT), and were maintained in indoor tanks of artificial seawater (ASW) (Marine Art BR; Tomita Pharmaceutical, Tokushima, Japan) at 18 °C. The adults were also collected from the pond on the Fukae campus of Kobe University, Kobe, Japan and from the fishing harbor in Murotsu, Hyogo, Japan. Eggs and sperm were obtained surgically from the gonoducts, and the eggs were fertilized in vitro. After insemination, the embryos were raised in ASW containing 50 µg/ml streptomycin sulfate (S6501; Sigma-Aldrich, St. Louis, MO, USA) at 18 °C.

### Preparation of reporter constructs and electroporation

Construction of the *vglut* > *kaede* was described previously^27,54^. The *vacht* > *cfp* plasmid was made by inserting the 3.8-kb upstream region of *Ciona vacht*^25^ into the *Sal*I/*Bam*HI site of pSP-CFP^54^. The 2.4-kb upstream region of *Ciona fer2* (Gene ID KH.L116.39) was previously cloned into the pSP-CFP vector^27^. The reporter sequence was replaced with a DNA fragment coding for mCherry to generate *fer2* > *mcherry* using *Not*I/*Eco*RI sites. The *gnrh2* > *kaede* and *gnrh2* > *mcherry* plasmids were made by inserting the 4.3-kb upstream region of *Ciona gnrh2*^12^ into the *Xho*I/*Not*I sites of the pSP-Kaede vector and pSP-mCherry vector, respectively^55^. The *gnrh2* upstream region was also used to generate the *gnrh2* > *g-camp8* construct. The Kaede coding sequence of pSP-Kaede was replaced with a DNA fragment coding for G-CaMP8^31^ using *Not*I/*Eco*RI sites. The *gnrh2* upstream region was amplified from the *gnrh2* > *kaede* plasmid using a pair of nucleotide primers (5′-GAATCGGCCAACGCGGGATCCAGGAGCAGACGTCATAAGTA-3′ and 5′-TGACGCGGCCGCTGTTACGTTATCTCTCTAGAAG-3′), digested with *Bam*HI and *Not*I, and then inserted into the *Bam*HI/*Not*I sites upstream of the G-CaMP8 in the pSP vector. Plasmid DNA constructs were electroporated into fertilized *Ciona* eggs as described by Corbo et al.^56^.

### Immunofluorescent staining

Immunofluorescent staining was carried out according to the method described by Nishitsuji et al.^22^. Photobleaching of fluorescent reporters, including CFP, mCherry, Kaede, and G-CaMP8, was not performed prior to immunofluorescent detection of these proteins. To avoid photoconversion of Kaede fluorescence from green to red, embryos and larvae transfected with the *kaede* transgene were kept away from short wavelength illumination during development, experiments, and observation, and no photoconversion was observed. All fluorescent images except those shown in Fig. 1 and Supplementary Fig. S1 were obtained by using a laser scanning confocal microscope (FV1200 IX83; Olympus, Tokyo) with a 40 × objective lens (numerical aperture (NA) 0.95; Olympus). The excitation/emission wavelengths for DAPI, Alexa Fluor 488, and Alexa Fluor 594 were 405 nm/461 nm, 473 nm/520 nm, and 559 nm/618 nm, respectively.

The fluorescent images shown in Fig. 1 and Supplementary Fig. S1 were obtained by using a fluorescent microscope (BX50; Olympus) with a 10 × objective lens (NA 0.40; Olympus) and a color fluorescence camera (DP74, Olympus). For these observations, the excitation and emission wavelengths were 470–490 nm and 515–550 nm, respectively.

To visualize the localization of cell type-specific proteins, a mouse antiserum against *Ciona* VIAAT^21^ or a rabbit antiserum against *Ciona* CRALBP^23^ was diluted 1:1000 in 10% goat serum in T-PBS (0.1% Triton X-100 in PBS) and used as the primary antibody. The secondary antibody was an Alexa Fluor 594-conjugated anti-mouse IgG (A11005; Thermo Fisher Scientific) or an Alexa Fluor 594-conjugated anti-rabbit IgG (A11012; Thermo Fisher Scientific).

The primary antibodies used to visualize the localization of fluorescent reporter proteins were rabbit anti-Kaede polyclonal (PM012; Medical & Biological Laboratories, Nagoya, Japan; for Kaede), rabbit anti-green fluorescent protein (GFP) polyclonal (A11122; Thermo Fisher Scientific; for G-CaMP8 and CFP), rat anti-red fluorescent protein (RFP) monoclonal (5F8; ChromoTek GmbH, Martinsried, Germany; for mCherry), and rat anti-GFP monoclonal (GF090R; Nacalai Tesque, Kyoto, Japan; for G-CaMP8 double-stained with anti-CRALBP) antibodies. All the primary antibodies were diluted 1000-fold as described above. The secondary antibodies were an Alexa Fluor 488-conjugated anti-rabbit IgG (A11008; Thermo Fisher Scientific) for G-CaMP8 and CFP, an Alexa Fluor 488-conjugated anti-rat IgG (A11006; Thermo Fisher Scientific) for G-CaMP8, and an Alexa Fluor 594-conjugated anti-rat IgG (A11007; Thermo Fisher Scientific) for mCherry.

### In vivo Ca^2+^ imaging

Electroporated *Ciona* larvae expressing the G-CaMP8 transgene were placed in ASW on a 35-mm glass-based dish (coverslip diameter 12 mm, #3931-035; Iwaki, Japan). For imaging, a microscope (IX81; Olympus, Tokyo) equipped with an electron multiplying charge-coupled device (EMCCD) camera (EVOLVE512; Photometrics, Tucson, AZ) and a 20 × objective lens (NA 0.80; Olympus) was used. Fluorescence excitation was done using a Spectra 4 LED light source (Lumencor, Beaverton, OR, USA) at 475 nm center wavelength. Images were taken through a band-pass emission filter (510–550 nm) with a 50-ms exposure time per 1 s and 1 × 1 binning. For each larva, 300 images were taken in 5 min. Changes in intracellular calcium concentrations were measured as the changes in the green fluorescence of G-CaMP8. The fluorescence intensity was spatially averaged in each region of interest (ROI). The fluorescence change was defined as Δ*F*/*F* = (*F*_*t*_ – *F*_*0*_)/*F*_*0*_, where *F*_*t*_ is the fluorescence intensity at time t, and *F*_*0*_ is the baseline averaged for 4–5 s. The fluorescence change (Δ*F*/*F*) was calculated after subtracting the background fluorescence. A MetaMorph image analysis software system (Molecular Devices) was used to analyze the images. Image processing was also performed with ImageJ (U. S. National Institutes of Health, Bethesda, MD, USA; https://imagej.nih.gov/ij/).

## Supplementary information

Supplementary Movie S1.

Supplementary Movie S2.

Supplementary Movie S3.

Supplementary Movie S4.

Supplementary Information.

## Data Availability

The authors confirm that the data supporting the findings of this study are available within the article and its supplementary materials and are available upon request.

## References

[1] Okubo K, Nagahama Y (2008). Structural and functional evolution of gonadotropin-releasing hormone in vertebrates. Acta Physiol..

[2] Oka Y (2009). Three types of gonadotrophin-releasing hormone neurones and steroid-sensitive sexually dimorphic kisspeptin neurones in teleosts. J. Neuroendocrinol..

[3] Dolan S, Evans NP, Richter TA, Nolan AM (2003). Expression of gonadotropin-releasing hormone and gonadotropin-releasing hormone receptor in sheep spinal cord. Neurosci. Lett..

[4] Albertson AJ, Talbott H, Wang Q, Jensen D, Skinner DC (2008). The gonadotropin-releasing hormone type I receptor is expressed in the mouse cerebellum. Cerebellum.

[5] Sherwood NM, Wu S (2005). Developmental role of GnRH and PACAP in a zebrafish model. Gen. Comp. Endocr..

[6] Wu S, Page L, Sherwood NM (2006). A role for GnRH in early brain regionalization and eye development in zebrafish. Mol. Cell. Endocrinol..

[7] Abraham E (2008). Early development of forebrain gonadotrophin-releasing hormone (GnRH) neurons and the role of GnRH as an autocrine migration factor. J. Neuroendocrinol..

[8] Kanaho YI (2009). Neurotrophic effect of gonadotropin-releasing hormone on neurite extension and neuronal migration of embryonic gonadotropin-releasing hormone neurons in chick olfactory nerve bundle culture. J. Neurosci. Res..

[9] Ramakrishnan S, Lee W, Navarre S, Kozlowski DJ, Wayne NL (2010). Acquisition of spontaneous electrical activity during embryonic development of gonadotropin-releasing hormone-3 neurons located in the terminal nerve of transgenic zebrafish (*Danio rerio*). Gen. Comp. Endocrinol..

[10] Delsuc F, Brinkmann H, Chourrout D, Philippe H (2006). Tunicates and not cephalochordates are the closest living relative of vertebrates. Nature.

[11] Putnam NH (2008). The amphioxus genome and the evolution of the chordate karyotype. Nature.

[12] Kusakabe TG (2012). A conserved non-reproductive GnRH system in chordates. PLoS ONE.

[13] Kamiya C (2014). Nonreproductive role of gonadotropin-releasing hormone in the control of ascidian metamorphosis. Dev. Dyn..

[14] Adams BA (2003). Six novel gonadotropin-releasing hormones are encoded as triplets on each of two genes in the protochordate, *Ciona intestinalis*. Endocrinology.

[15] Kusakabe T, Mishima S, Shimada I, Kitajima Y, Tsuda M (2003). Structure, expression, and cluster organization of genes encoding gonadotropin-releasing hormone receptors found in the neural complex of the ascidian *Ciona intestinalis*. Gene.

[16] Tello JA, Rivier JE, Sherwood NM (2005). Tunicate gonadotropin-releasing hormone (GnRH) peptides selectively activate *Ciona intestinalis* GnRH receptors and the green monkey type II GnRH receptor. Endocrinology.

[17] Sakai T, Aoyama M, Kusakabe T, Tsuda M, Satake H (2010). Functional diversity of signaling pathways through G protein-coupled receptor heterodimerization with a species-specific orphan receptor subtype. Mol. Biol. Evol..

[18] Sakai T (2012). Evidence for differential regulation of GnRH signaling via heterodimerization among GnRH receptor paralogs in the protochordate,* Ciona intestinalis*. Endocrinology.

[19] Abitua PB (2015). The pre-vertebrate origins of neurogenic placodes. Nature.

[20] Horie T, Kusakabe T, Tsuda M (2008). Glutamatergic networks in the *Ciona intestinalis* larva. J. Comp. Neurol..

[21] Horie T, Nakagawa M, Sasakura Y, Kusakabe TG, Tsuda M (2010). Simple motor system of the ascidian larva: neuronal complex comprising putative cholinergic and GABAergic/glycinergic neurons. Zool. Sci..

[22] Nishitsuji K (2012). Cell lineage and *cis*-regulation for a unique GABAergic/glycinergic neuron type in the larval nerve cord of the ascidian *Ciona intestinalis*. Dev. Growth. Differ..

[23] Tsuda M (2003). Origin of the vertebrate visual cycle. II. Visual cycle proteins are localized in whole brain including photoreceptor cells of a primitive chordate. Vis. Res..

[24] Kusakabe TG, Takimoto N, Jin M, Tsuda M (2009). Evolution and the origin of the visual retinoid cycle in vertebrates. Philos. Trans. R. Soc. Lond. B Biol. Sci..

[25] Yoshida R (2004). Identification of neuron-specific promoters in *Ciona intestinalis*. Genesis.

[26] Katz MJ (1983). Comparative anatomy of the tunicate tadpole,* Ciona intestinalis*. Biol. Bull..

[27] Razy-Krajka F (2012). Monoaminergic modulation of photoreception in ascidian: evidence for a proto-hypothalamo-retinal territory. BMC Biol..

[28] Moret F (2005). The dopamine-synthesizing cells in the swimming larva of the tunicate *Ciona intestinalis* are located only in the hypothalamus-related domain of the sensory vesicle. Eur. J. Neurosci..

[29] Horie T (2018). Regulatory cocktail for dopaminergic neurons in a protovertebrate identified by whole-embryo single-cell transcriptomics. Genes Dev..

[30] Gyoja F, Satoh N (2013). Evolutionary aspects of variability in bHLH orthologous families: Insights from the pearl oyster, *Pinctada fucata*. Zool. Sci..

[31] Ohkura M (2012). Genetically encoded green fluorescent Ca^2+^ indicators with improved detectability for neuronal Ca^2+^ signals. PLoS ONE.

[32] Nishino A, Okamura Y, Piscopo S, Brown ER (2010). A glycine receptor is involved in the organization of swimming movements in an invertebrate chordate. BMC Neurosci..

[33] Kusakabe TG, Shigeno S, Murakami Y, Nomura T (2017). Identifying vertebrate brain prototypes in deuterostomes. Brain evolution by design: from neural origin to cognitive architecture.

[34] Nishino A, Baba SA, Okamura Y (2011). A mechanism for graded motor control encoded in the channel properties of the muscle ACh receptor. Proc. Natl. Acad. Sci. USA.

[35] Hackley C, Mulholland E, Kim GJ, Newman-Smith E, Smith WC (2013). A transiently expressed connexin is essential for anterior neural plate development in *Ciona intestinalis*. Development.

[36] Abdul-Wajid S, Morales-Diaz H, Khairallah SM, Smith WC (2015). T-type calcium channel regulation of neural tube closure and EphrinA/EPHA expression. Cell Rep..

[37] Akahoshi T, Hotta K, Oka K (2017). Characterization of calcium transients during early embryogenesis in ascidians *Ciona robusta* (*Ciona intestinalis* type A) and *Ciona savignyi*. Dev. Biol..

[38] Gu X, Olson EC, Spitzer NC (1994). Spontaneous neuronal calcium spikes and waves during early differentiation. J. Neurosci..

[39] Wong RO, Chernjavsky A, Smith SJ, Shatz CJ (1995). Early functional neural networks in the developing retina. Nature.

[40] Feller MB, Wellis DP, Stellwagen D, Werblin FS, Shatz CJ (1996). Requirement for cholinergic synaptic transmission in the propagation of spontaneous retinal waves. Science.

[41] Zhou ZJ (1998). Direct participation of starburst amacrine cells in spontaneous rhythmic activities in the developing mammalian retina. J. Neurosci..

[42] Kerr JN, Greenberg D, Helmchen F (2005). Imaging input and output of neocortical networks in vivo. Proc. Natl. Acad. Sci. USA.

[43] Chang LW, Spitzer NC (2009). Spontaneous calcium spike activity in embryonic spinal neurons is regulated by developmental expression of the Na^+^, K^+^-ATPase β3 subunit. J. Neurosci..

[44] Feller MB (1999). Spontaneous correlated activity in developing neural circuits. Neuron.

[45] Spitzer NC, Lautermilch NJ, Smith RD, Gomez TM (2000). Coding of neuronal differentiation by calcium transients. BioEssays.

[46] Nakagawa M, Miyamoto T, Ohkuma M, Tsuda M (1999). Action spectrum for the photophobic response of *Ciona intestinalis* (Ascidiacea, Urochordata) larvae implicates retinal protein. Photochem. Photobiol..

[47] Tsuda M, Kawakami I, Shiraishi S (2003). Sensitization and habituation of the swimming behavior in ascidian larvae to light. Zool. Sci..

[48] Zega G, Thorndyke MC, Brown ER (2006). Development of swimming behavior in the larva of the ascidian *Ciona intestinalis*. J. Exp. Biol..

[49] Reist NE, Smith SJ (1992). Neurally evoked calcium transients in terminal Schwann cells at the neuromuscular junction. Proc. Natl. Acad. Sci. USA.

[50] Newman EA, Zahs KR (1997). Calcium waves in retinal glial cells. Science.

[51] Robitaille R (1998). Modulation of synaptic efficacy and synaptic depression by glial cells at the frog neuromuscular junction. Neuron.

[52] Haydon PG (2001). Glia: listening and talking to the synapse. Nat. Rev. Neurosci..

[53] Fenno L, Yizhar O, Deisseroth K (2011). The development and application of optogenetics. Ann. Rev. Neurosci..

[54] Horie T (2011). Ependymal cells of chordate larvae are stem-like cells that form the adult nervous system. Nature.

[55] Hozumi A (2010). Efficient transposition of a single *Minos* transposon copy in the genome of the ascidian *Ciona intestinalis* with a transgenic line expressing transposase in eggs. Dev. Dynam..

[56] Corbo JC, Levine M, Zeller RW (1997). Characterization of a notochord-specific enhancer from the *Brachyury* promoter region of the ascidian, Ciona intestinalis. Development.

