# Supplementary material for: Cellular identity and Ca2+ signaling activity of the non-reproductive GnRH system in the Ciona intestinalis type A (Ciona robusta) larva
Source: Sci Rep. 2020 Oct 29;10:18590. doi: 10.1038/s41598-020-75344-7 (PMC7596717; doi:10.1038/s41598-020-75344-7)
Supplement: Supplementary file 5 — Supplementary Information. [file 41598_2020_75344_MOESM5_ESM.pdf]

Cellular identity and Ca^{2+} signaling activity of the non-reproductive GnRH system in the *Ciona intestinalis* type A (*Ciona robusta*) larva

Supplementary Figures

Nanako Okawa[1,2], Kotaro Shimai[1,2], Kohei Ohnishi[1,2], Masamichi Ohkura[3], Junichi Nakai[3,4], Takeo Horie[5], Atsushi Kuhara[1,2], and Takehiro G. Kusakabe[1,2,*]

[1] Department of Biology, Faculty of Science and Engineering, Konan University, 8-9-1 Okamoto, Higashinada-ku, Kobe 658-8501, Japan
[2] Institute for Integrative Neurobiology, Graduate School of Natural Science, Konan University, 8-9-1 Okamoto, Higashinada-ku, Kobe 658-8501, Japan
[3] Brain and Body System Science Institute, Saitama University, Saitama, Japan
[4] Brain Division of Oral Physiology, Department of Oral Function and Morphology, Tohoku University Graduate School of Dentistry, 4-1 Seiryo-machi, Aoba-ku, Sendai 980-8575, Japan
[5] Shimoda Marine Research Center, University of Tsukuba, Shimoda, 415-0025, Japan

*Corresponding author: tgk@konan-u.ac.jp

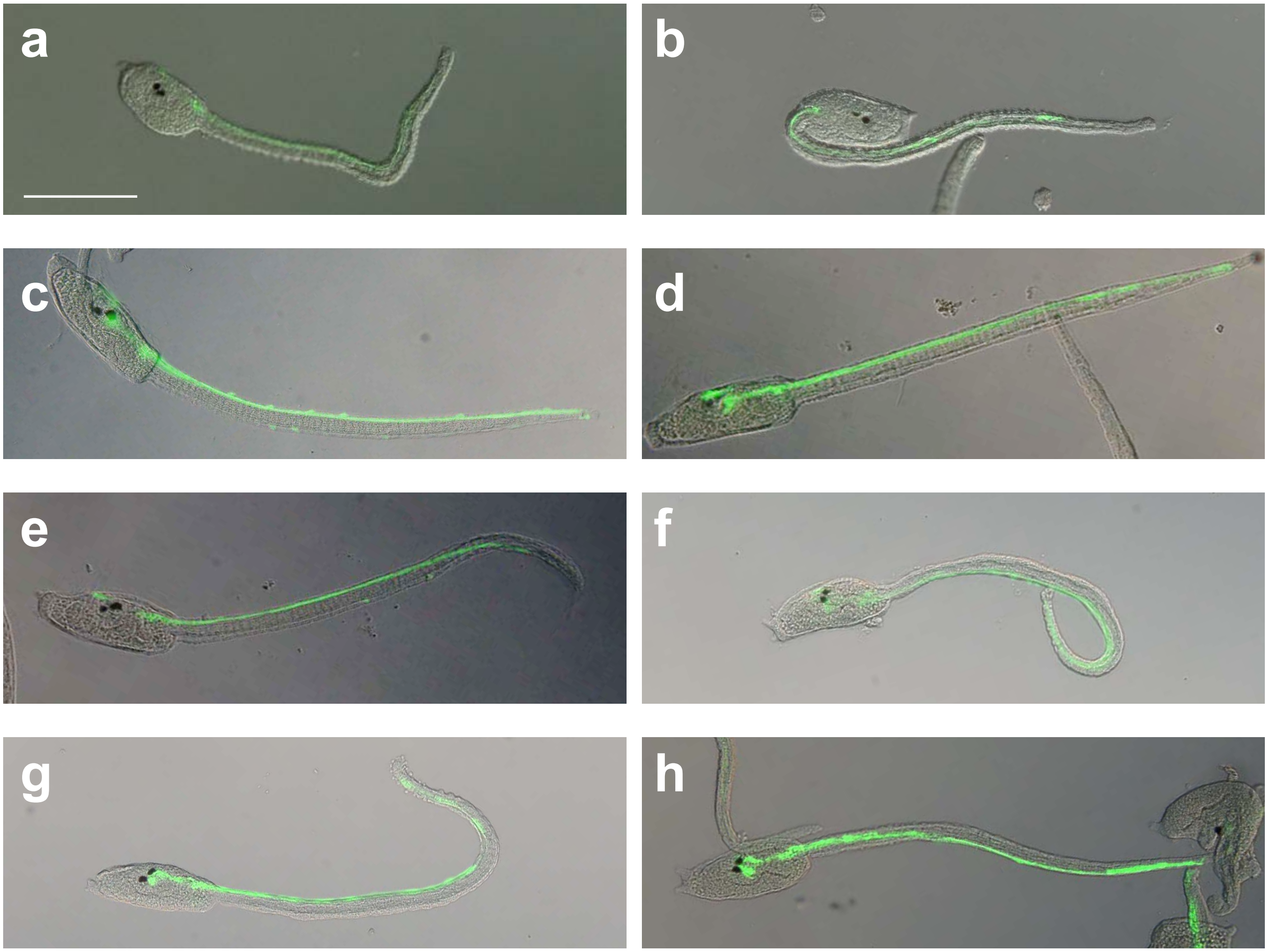

Figure S1. Examples of the expression patterns of *gnrh2>g-camp8* at various larval stages. Larvae developed from eggs electroporated with *gnrh2>g-camp8* were fixed at 19 hpf **(a,b)**, 21 hpf **(c–e)**, and 23 hpf **(f–h)**, and localization of G-CaMP8 was visualized by immunofluorescent staining. Expression patterns were quite similar between different larval stages. Scale bar, 200 μm.

Okawa, N. et al. Figure S2

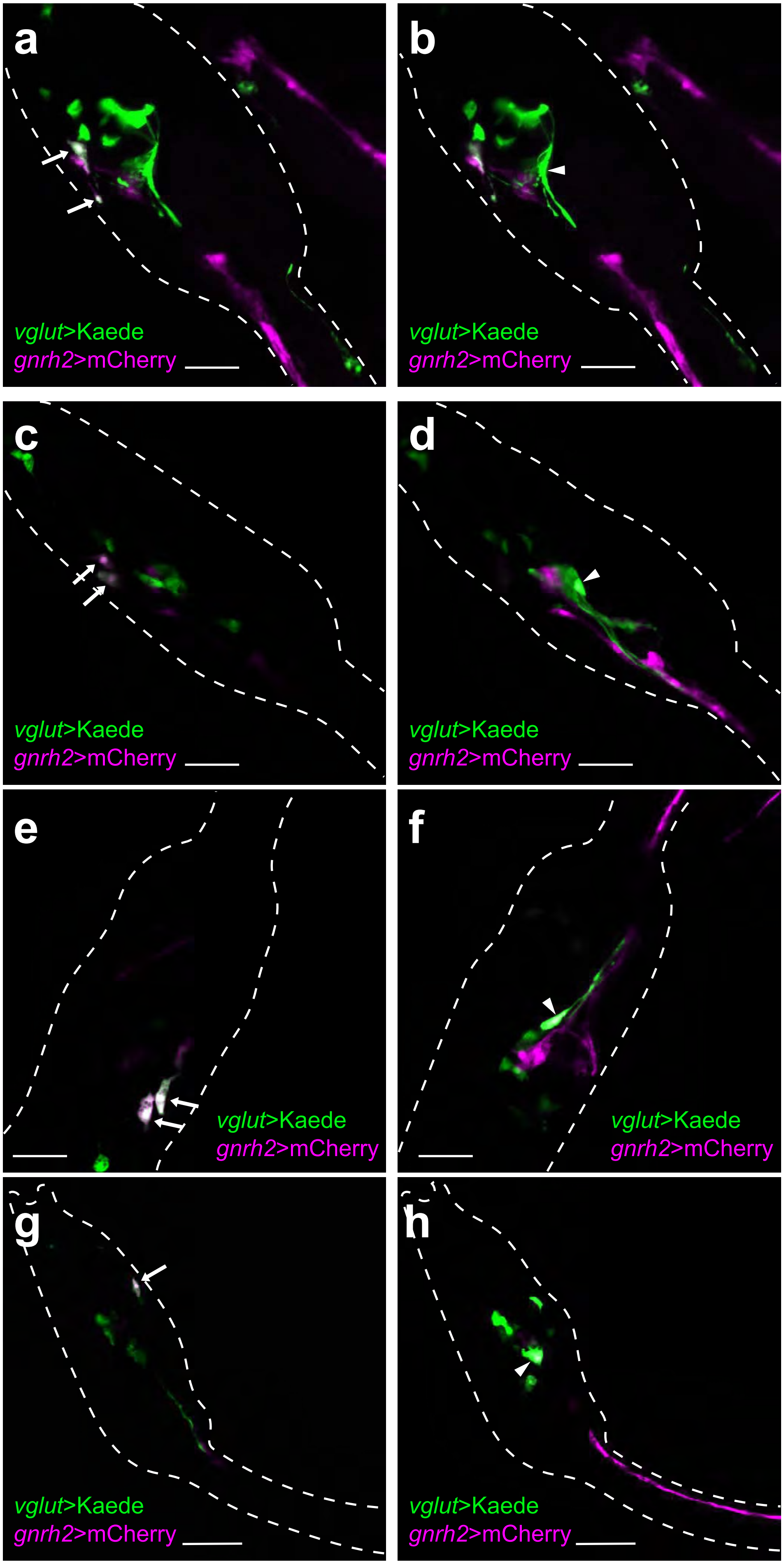

Figure S2. Double immunofluorescent staining of cells expressing *gnrh2* and glutamatergic neurons in the *Ciona* larva at 21 hpf (a–f) and 22.5 hpf (g,h). In each row, the left and right panels are different optical sections from the same larva, showing aATENs (*arrows*) and glutamatergic posterior brain vesicle neurons (*arrowheads*), respectively. Glutamatergic neurons and *gnrh2*-expressing cells were labeled with Kaede (*green*) and mCherry (*magenta*), respectively. In the larvae shown in **(f)** and **(h)**, Kaede and mCherry signals were overlapped in the posterior brain vesicle (*arrowheads*). **(a)** Composite confocal image of three optical sections (section nos. 12, 19, and 25 among 47 serial sections taken at 0.60 μm intervals). **(b)** Projection of 5 serial optical sections taken at 0.60 μm intervals. **(c)** Composite confocal image of two optical sections (section nos. 7 and 12 among 33 serial sections taken at 0.60 μm intervals). **(d)** Projection of 3 serial optical sections taken at 0.60 μm intervals. **(e)** Confocal image of a single optical section. **(f)** Projection of 3 serial optical sections taken at 0.60 μm intervals. **(g)** Confocal image of a single optical section. **(h)** Projection of 3 serial optical sections taken at 0.60 μm intervals.

Okawa, N. et al. Figure S3

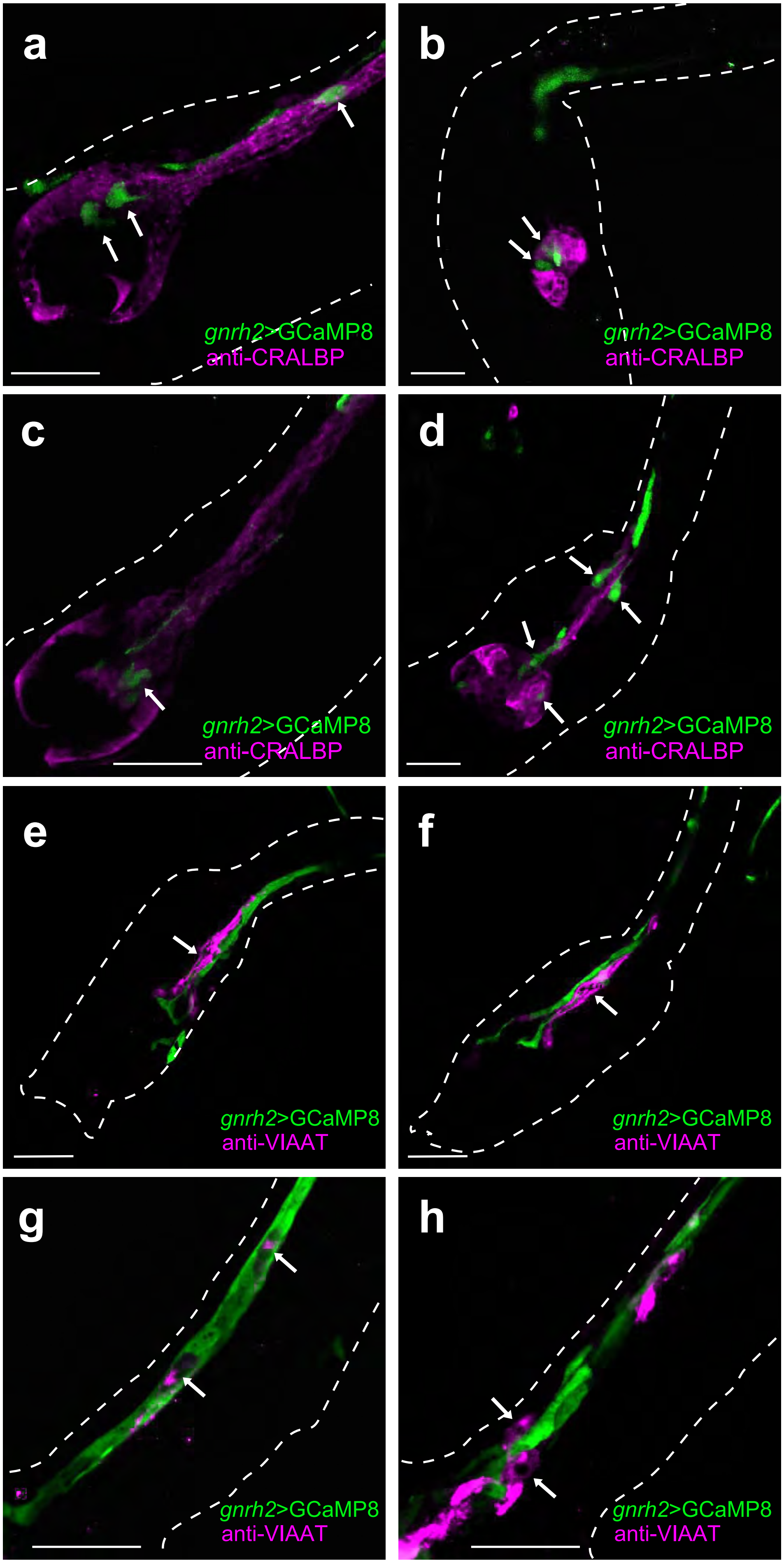

Figure S3. CRALBP-positive cells and GABAergic/glycinergic neurons seem not to express *gnrh2*. (a–d) Double immunofluorescent staining of cells expressing *gnrh2* and CRALBP-positive cells in the brain vesicle and motor ganglion of *Ciona* larvae at 21 hpf. CRALBP-positive cells (*magenta*) were not overlapped with *gnrh2*-expressing cells (*green*). *Arrows* indicate *gnrh2*-expressing cells in the brain vesicle. **(e–h)** GABAergic/glycinergic neurons were visualized by immunostaining with anti-VIAAT antibody (*magenta*) in larvae at 21 hpf. *Arrows* in **(e,f)** indicate VIAAT-positive neurons in the motor ganglion. *Arrows* in **(g,h)** indicate VIAAT-positive ACINs. Projections of serial optical sections taken at 0.60 μm intervals; the number of sections stacked were 5 **(a,b)**, 6 **(c)**, 3 **(d–e)**, 2 **(g)**, and 4 **(h)**. Scale bars, 30 μm.

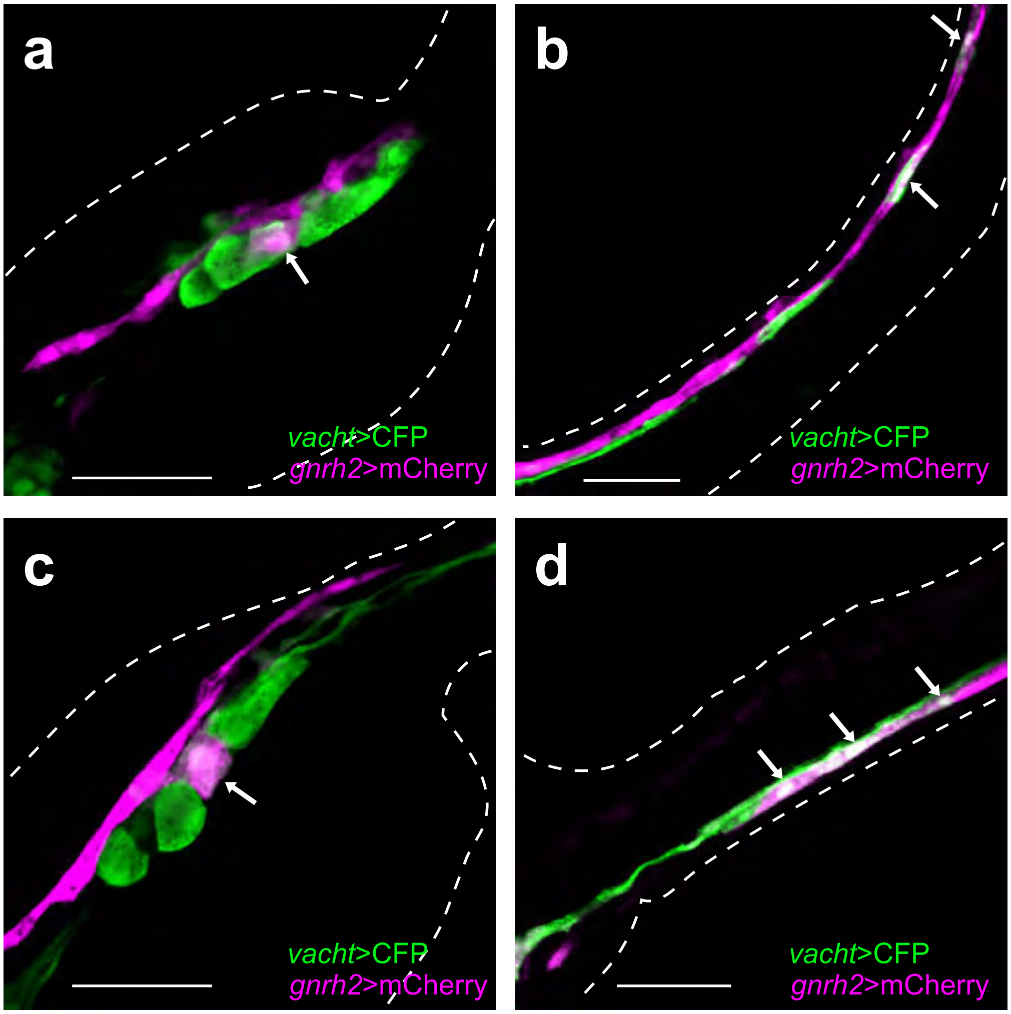

Figure S4. Some cholinergic neurons express *gnrh2*. Cholinergic neurons and *gnrh2*-exressing cells were labeled with CFP (*green*) and mCherry (*magenta*), respectively. *Arrows* indicate cells that co-expressed both markers. Overlapped signals were observed in neurons in the motor ganglion **(a,c)** and neurons along the nerve cord **(b,d)**. **(a)** Projection of 5 serial optical sections taken at 0.60 μm intervals. **(b)** Projection of 3 serial optical sections taken at 0.60 μm intervals. **(c,d)** Confocal image of a single optical section. Scale bars, 30 μm.

Okawa, N. et al. Figure S5

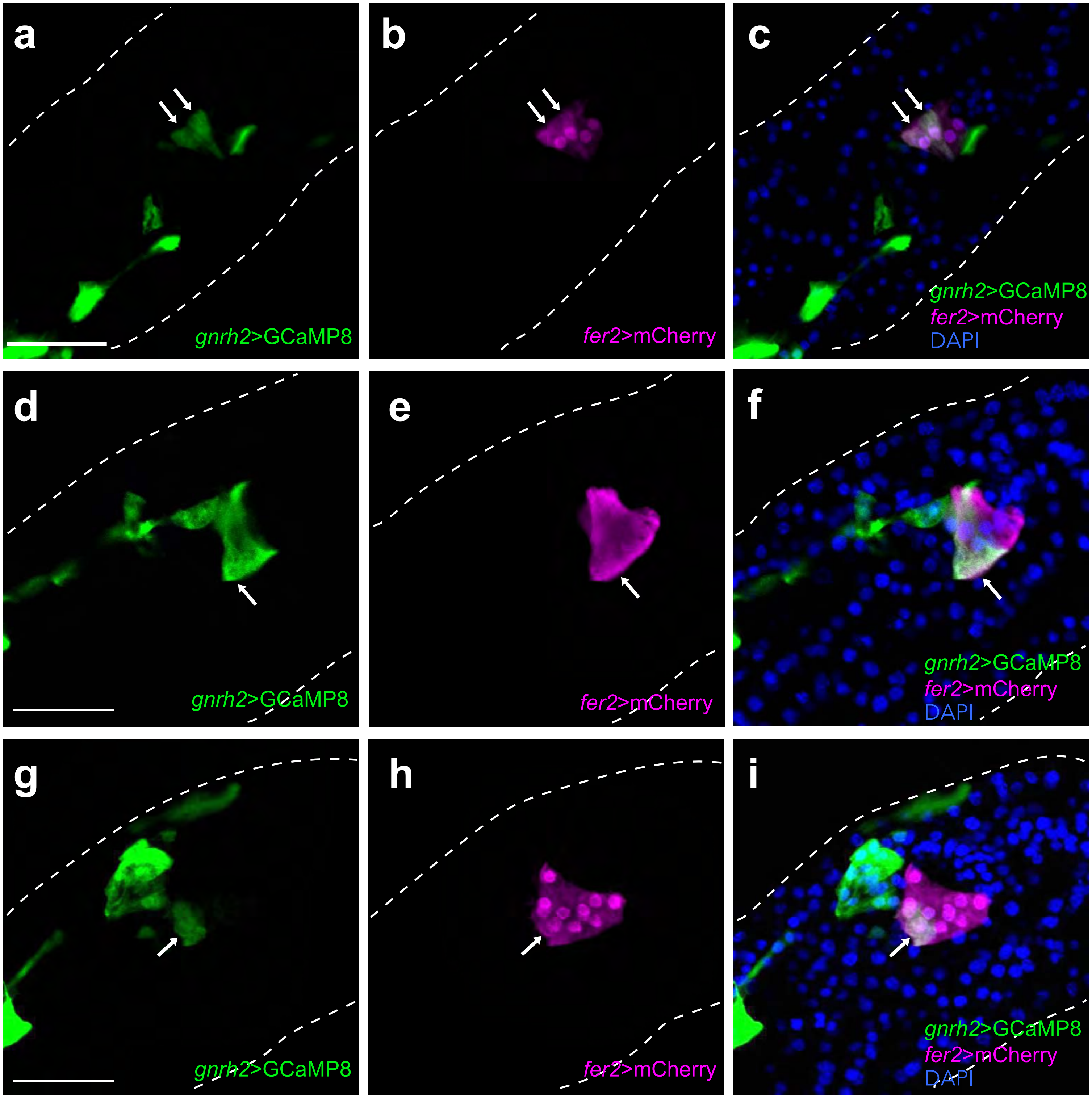

Figure S5. Dopaminergic cells and *gnrh2*-expressing cells were labeled with mCherry (*magenta*) and G-CaMP8 (*green*), respectively. Left, middle, and right panels show G-CaMP8, mCherry, and merged signals, respectively. **(a–c)** The same larva as shown in **Fig. 3c**. Confocal image of a single optical section. **(d–f)** Confocal image of a single optical section. **(g–i)** Projection of 7 serial optical sections taken at 0.45 μm intervals. Scale bars, 30 μm.

Okawa, N. et al. Figure S6

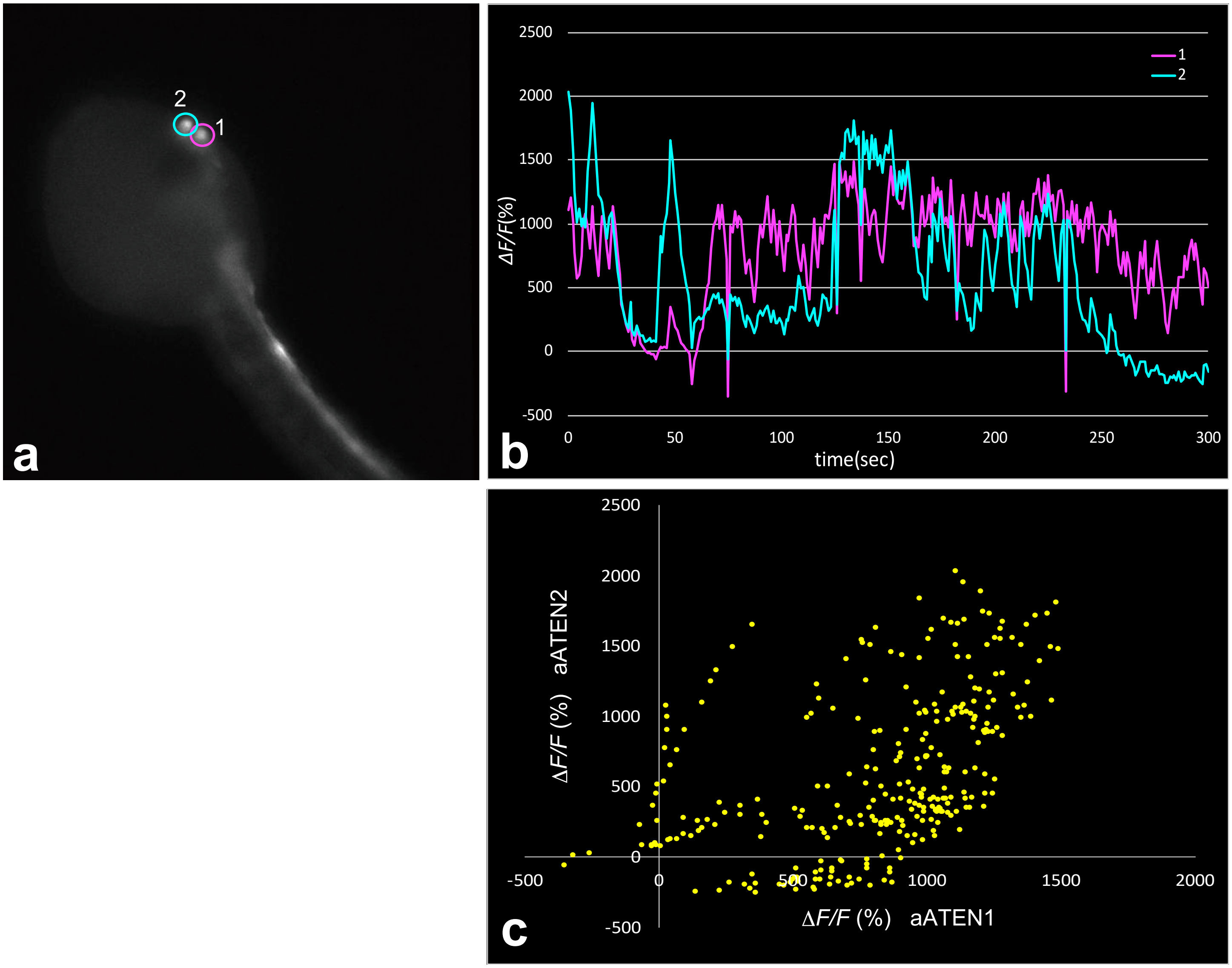

Figure S6. Detailed characteristics of Ca^{2+} transients in the aATENs in the larva shown in Figure 4b. (a) Fluorescence image of a larva at 19 hpf, showing G-CaMP8 fluorescence in the pair of aATENs (numbered circles). **(b)** The graph shows the temporal patterns of fluorescence intensity in the aATENs indicated by circles in **(a)**. **(c)** The scatter plot shows the relationship between G-CaMP8 fluorescence in the two aATENs at each time point shown in **(b)**. The correlation coefficient is 0.478.

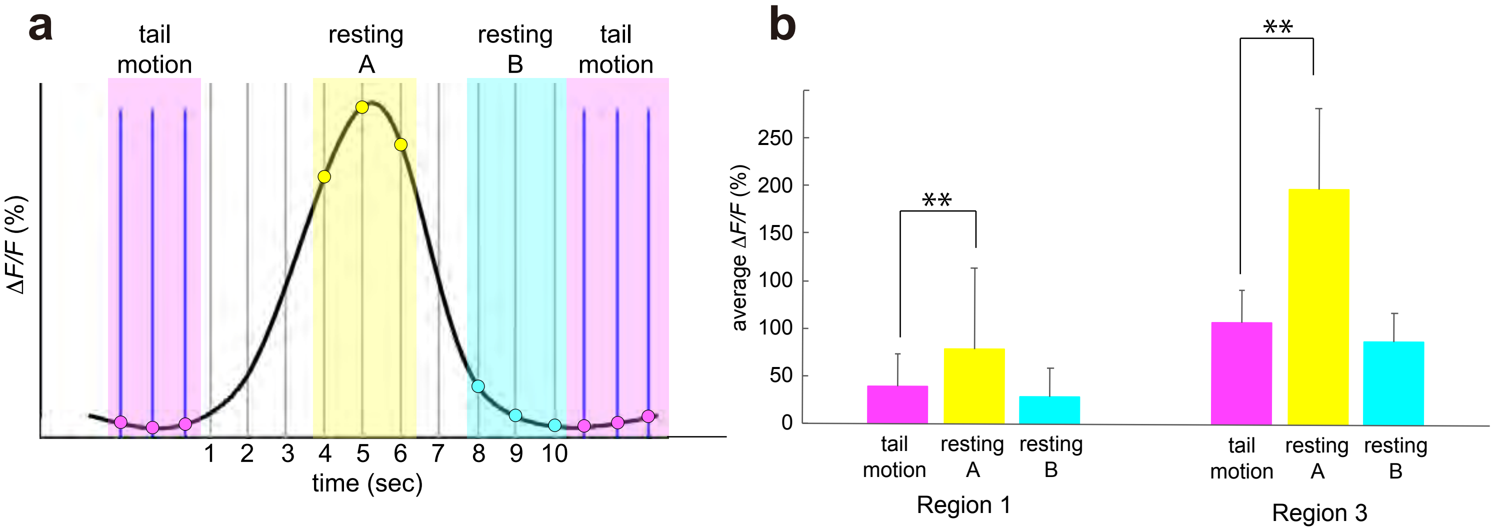

Figure S7. Evaluation of the relationship between Ca^{2+} transients and tail movement in the larva shown in Figure 7. (a) G-CaMP8 fluorescence intensity was compared between the periods when the tail was moving (designated as 'tail motion') and the two different time windows during the period the tail was not moving (designated as 'resting A' and 'resting B'). A 'resting A' period included three exposure time points starting from the fourth exposure time after a 'tail motion' period. A 'resting B' period included three exposure time points immediately before the next 'tail motion' period. Only when the interval between two 'tail motion' periods contains at least nine exposure time points are subjected to the analysis so that a 'resting A' period and the succeeding 'resting B' period did not overlap. **(b)** Comparison of the average fluorescence intensity between 'tail motion' periods and the 'resting A' or 'resting B' periods. Region 1 and Region 3 correspond to ROI #1 and #3 in **Figure 7b**, respectively. Statistical analysis was carried out using the standard Student t-test (*$**P < 0.01$*).
